# Supplementary material for: Influence of pyrethroïd-treated bed net on host seeking behavior of Anopheles gambiae s.s. carrying the kdr allele
Source: PLoS One. 2017 Jul 31;12(7):e0164518. doi: 10.1371/journal.pone.0164518 (PMC5536278; doi:10.1371/journal.pone.0164518)
Supplement: S1 Table — (PDF) [file pone.0164518.s001.pdf]

S1 Table: Effect of treatment on environmental variables. Comparison between the two arms. ***p<0.001, **p≤0.01, *p≤0.05, ns= not significant.

Treatment	Environmental variable	Significant
Empty	CO2	ns
	HR	ns
Rabbit alone	CO2	ns
	HR	ns
Rabbit+UTN	CO2	ns
	HR	**
Rabbit+ITN	CO2	ns
	HR	ns
